# Orbital Inflammatory Complications of Crohn’s Disease: A Rare Case Series

**DOI:** 10.1177/1179552218757512

**Published:** 2018-02-20

**Authors:** Tanya M Monaghan, Giorgio Albanese, Philip Kaye, James D Thomas, Lorraine C Abercrombie, Gordon W Moran

**Affiliations:** 1 Nottingham Digestive Diseases Centre, National Institute for Health Research (NIHR) Nottingham Biomedical Research Centre, Nottingham University Hospitals NHS Trust and University of Nottingham, Nottingham, UK; 2 Department of Ophthalmology, Nottingham University Hospitals NHS Trust, Nottingham, UK; 3 Department of Cellular Pathology, Nottingham University Hospitals NHS Trust, Nottingham, UK; 4 Department of Radiology, Nottingham University Hospitals NHS Trust, Nottingham, UK

**Keywords:** Orbital inflammation, inflammatory bowel disease

## Abstract

Orbital inflammatory disease is a rare ophthalmic manifestation of Crohn’s disease. Inflammation is characteristically nonspecific, involving one or multiple structures of the orbit. Mechanisms of disease and optimal methods of treatment are poorly understood. The aim of this report is to present 3 cases of orbital involvement in Crohn’s disease. A retrospective case note review of patients with orbital inflammatory disease and Crohn’s disease was performed at our academic center to determine the clinical, imaging, and histopathologic features of this condition and its relationship to intestinal Crohn’s disease. Three patients were identified with orbital inflammatory manifestations complicating Crohn’s disease. All patients described were women with active intestinal disease and had a history of treatment with immunosuppressive therapies. Similarities were observed in clinical presentations with variance noted in radiologic and histopathologic findings. In all cases, symptoms improved with oral corticosteroids or nonsteroidal drugs in combination with anti-tumor necrosis factor agents. Inflammatory bowel disease–related orbital complications are rare but potentially vision-threatening. It is important to consider mimics of orbital inflammatory disease such as systemic inflammatory disease, malignancy, congenital malformations, infection, and trauma when formulating a comprehensive differential diagnosis. Therapeutic intervention is directed toward preservation of vision and orbital function and reducing the acute inflammatory process. Corticosteroids are typically the initial treatment of choice for moderate-to-severe disease, although several classes of immunomodulatory agents have been variably useful in treating this condition. Heightened awareness and close cooperation between gastroenterologists and ophthalmologists are mandatory.

## Introduction

The reported incidence of ocular manifestations in patients with inflammatory bowel disease (IBD) is variable, ranging from 0.3% to 13%, 1.6% to 5.4% among those with ulcerative colitis and 3.5% to 6.8% among those with Crohn’s disease,^1^ and may be primary, secondary to treatment, effects of the intestinal disease or coincidental.^2,3^ Ocular complications occur more frequently in patients with Crohn’s disease,^4,5^ rather than ulcerative colitis, and an association has been reported with female sex.^1,5^ Patients with a colonic or ileocolonic disease location tend to have a higher incidence of ocular involvement compared with those with only a small bowel disease location.^6,7^ The presence of other extraintestinal manifestations (EIMs) such as arthralgia in Crohn’s disease has been associated with ocular involvement.^8^ Although episcleritis, scleritis, and uveitis are the most common ocular-EIMs (O-EIMs) in IBD,^2^ orbital inflammatory disease (OID) has also been reported as a rare EIM. Orbital inflammatory disease can present with an array of findings depending on the structures affected by the combined effects of inflammation, elevated orbital pressure, and direct compression. Common findings include conjunctival injection, chemosis, eyelid swelling, proptosis, diplopia, pain with eye movement, ophthalmoplegia, and impaired vision.^3^ Important differentials to consider in OID include malignancies, congenital mass lesions, orbital cellulitis, and occult or distant trauma. Systemic inflammatory diseases associations of OID include autoimmune thyroid disease, sarcoidosis, granulomatosis with polyangiitis (GPA), systemic lupus erythematosus, and other connective tissue diseases.^9^ Dacryoadenitis (localized inflammation of the lacrimal gland) is the rarest form of ocular adnexal involvement in IBD and typically presents with unilateral upper lid swelling, erythema, and pain in the superotemporal orbit.^10–12^ Herein, we report a rare case series of 3 female patients with Crohn’s disease developing heterogeneous manifestations of OID.

## Case History 1

**Figure 1.**
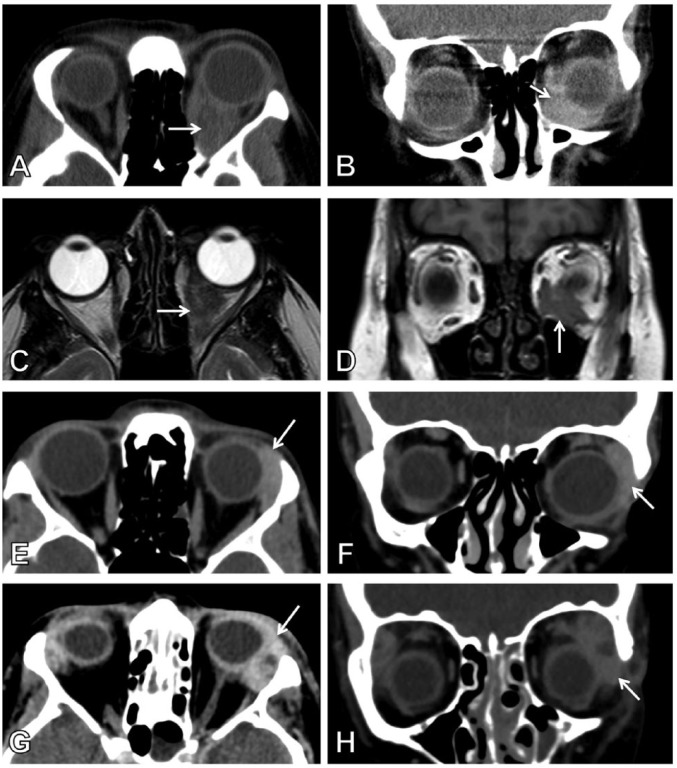
Orbital imaging in the 3 cases. Arrows indicate the site of the inflammatory masses. (A, B) Axial and coronal CT reformats of case 1. (C, D) Axial and coronal MRI of case 1. (E, F) Axial and coronal CT reformats of case 2. (G, H) Axial and coronal CT reformats of case 3. CT indicates computed tomography; MRI, magnetic resonance imaging.

A 48-year-old woman was diagnosed with ileocolonic nonstricturing, nonpenetrating Crohn’s disease at the age of 41 years. She described a 7-year history of joint pains and recurrent iritis preceding her diagnosis. Her prior treatments included 6-mercaptopurine, adalimumab, methotrexate, and a polymeric diet. While on a polymeric diet only due to previous drug intolerance issues and having stopped methotrexate 2 weeks prior to her presentation, she presented with a 2-month history of left lower eyelid swelling, diplopia, and ptosis. An orbital mass was palpable inferiorly which was causing elevation of the globe with reduced movement. Blood analysis revealed a raised white cell count (WCC) of 16 × 10^9^/L and a C-reactive protein (CRP) of 300 mg/L. Computed tomography (CT) and subsequent magnetic resonance imaging of the orbits revealed a well-defined lesion in the inferior aspect of the left orbit, involving the inferior rectus, without any other orbital abnormality (Figure 1A to D). Histopathologic assessment showed multiple noncaseating granulomas, some of which contained multinucleate giant cells (Figure 3A). Immunostaining was negative for Ziehl-Neelsen and IgG4. The patient was commenced on a 4-week reducing course of oral prednisolone, starting dose of 40 mg with recommencement of weekly 15 mg subcutaneous methotrexate. After 2 weeks of discharge, she was reviewed and noted to have made a full recovery with no recurrence of her luminal or orbital symptoms.

## Case History 2

**Figure 2.**
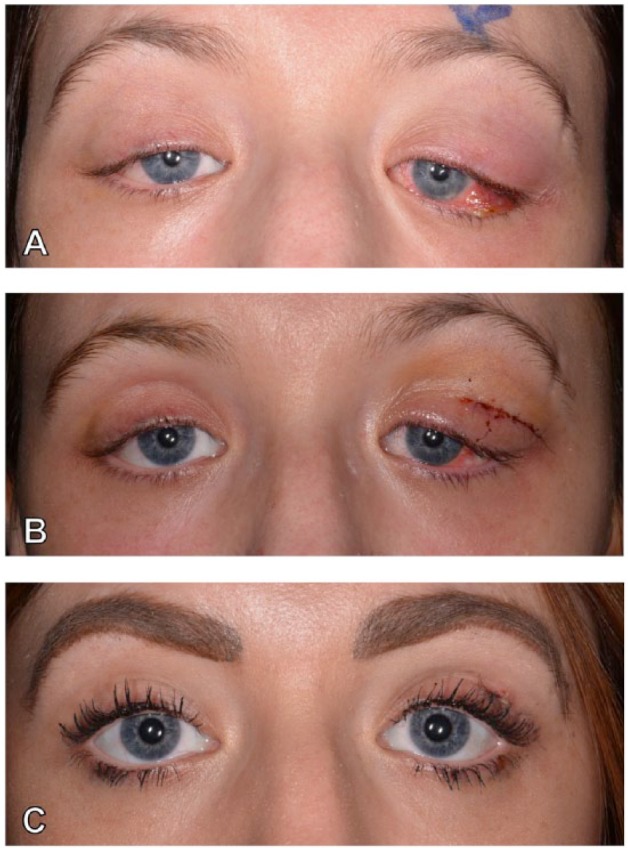
Photographs of the patient in case 2 demonstrating the presenting signs and postoperative recovery. (A) At diagnosis, (B) immediately postoperatively, and (C) patient at 1-week post-discharge.

**Figure 3.**
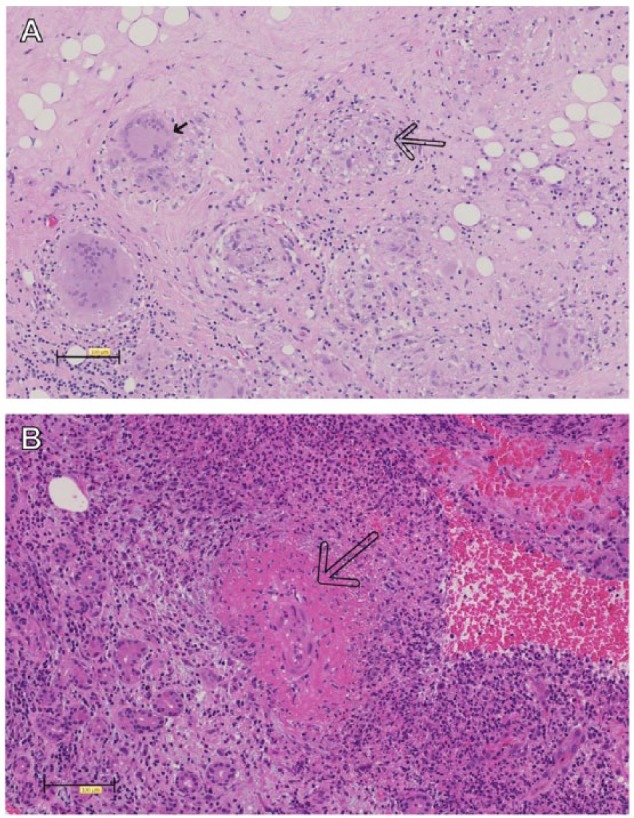
Representative histology using haematoxylin and eosin stain. (A) Orbital granuloma and (B) lacrimal granuloma with prominent vasculitis. Small arrow indicates granuloma, large outline arrow indicates vasculitis.

A 27-year-old woman was diagnosed with ileocolonic nonstricturing, nonpenetrating Crohn’s disease at the age of 17 years. Other comorbidities included osteopenia, previous erythema nodosum, sinusitis, and a positive lupus anticoagulant result. Her family history included autoimmune hemolytic anemia, GPA, and tuberculosis. In the 4 years following diagnosis, her Crohn’s disease followed a steroid-refractory course complicated by stricturing of the large intestine, leading to a segmental transverse colon resection and right-sided double-barrelled colostomy. After 1 year, she underwent a loop ileostomy for a perforated internal hernia. In the subsequent 5 years, additional surgeries were also performed for reversal of the loop ileostomy and subsequent refashioning of the colostomy. Her treatments included several courses of corticosteroids, azathioprine (developed intolerance), infliximab (anaphylaxis), and later adalimumab with addition of methotrexate. While off adalimumab for 1 week (recurrent viral infections), she presented with a 2-day history of acute onset of bilateral painful S-shaped periorbital swelling, moderate erythema, and chemosis worse on the left with diplopia (Figure 2A). Ocular movements were restricted and episcleritis was evident. A CT scan of head, orbits, and sinuses revealed bilateral lacrimal gland enlargement. Lateral and superior recti were also enlarged (Figure 1E to F). Blood analysis showed raised inflammatory markers with a WCC of 21.6 × 10^9^/L and a CRP of 166 mg/L, normal serum angiotensin-converting enzyme (ACE) levels, and thyroid function. Immunologic tests showed positive perinuclear antineutrophil cytoplasmic antibodies (p-ANCAs) and low proteinase 3 (PR3). Neither pulmonary nor renal involvement was present. Postoperative appearances are shown in Figure 2B. Histology showed a chronic dacryoadenitis, acute vasculitis of medium-sized vessels, and necrotizing histiocytic granulomas (Figure 3B). Stains for periodic acid-Schiff-diastase, Ziehl-Neelsen, and IgG4 were negative, and pus collected from the lacrimal gland during the biopsy did not reveal any microbial growth. The patient was started on a 2-day course of intravenous Co-amoxiclav (1.2 g 3 times daily) and an 8-week reducing course of high-dose oral prednisolone (commencing at 60 mg daily) for presumed OID complicating Crohn’s disease. Following recommencement of weekly 25 mg subcutaneous methotrexate and biweekly adalimumab, her symptoms and signs had completely resolved at ophthalmological assessment 1 week later (Figure 2C).

## Case History 3

A 33-year-old woman was diagnosed with colonic nonstricturing, nonpenetrating Crohn’s disease at the age of 21 years. Comorbidities included previous sinusitis. The patient was treated with azathioprine and subsequently infliximab 5 mg/kg every 7 weeks. Recently, she had experienced a relapse of her Crohn’s disease symptoms with endoscopic findings in keeping with active left-sided colonic disease. The patient presented with a 4-month history of left-sided swelling in the lacrimal gland area with typical S-shaped configuration, which had become more obvious over the preceding few days prior to hospitalization. On examination, there was an S-shaped deformity of the left upper eyelid along with mild eyelid edema and a lobulated swelling of the lacrimal gland. Computed tomography of the orbits showed an enlarged left lacrimal gland, which enhanced uniformly post-contrast (Figure 1G and H). Blood analysis showed a raised CRP of 55 mg/L. Histology revealed a dacryoadenitis featuring numerous, well-circumscribed noncaseating granulomas, some of which contained multinucleate giant cells. Special stains for microorganisms (Gram, Grocott, periodic acid-Schiff, and Ziehl-Neelsen) and IgG4 were negative. The presence of dacryoadenitis in the absence of a raised serum ACE level favored a diagnosis of an extraintestinal manifestation of Crohn’s disease rather than sarcoidosis. The patient was commenced on a 2-day course of 100 mg twice daily flurbiprofen due to a previous psychotic reaction to corticosteroids followed by infliximab. Subsequent follow-up revealed complete resolution of symptoms aside from some residual scar tissue at the biopsy site.

## Discussion

We report a case series of OID developing in 3 immunosuppressed female patients with active Crohn’s disease (Table 1). Orbital inflammatory disease is a rare EIM of IBD. Consequently, its exact association with clinical characteristics of the intestinal disease and patient demographics is uncertain.^5^ In a review of 24 patients with biopsy-proven nonspecific OID, the lacrimal gland was affected in 54.2%, extraocular muscles 50%, orbital fat 75%, sclera 4.2%, optic nerve 20.8%, and other structures in 8.3%.^13^ The histopathologic spectrum of nonspecific OID is typically nondiagnostic, secondary to a wide range of presentations ranging from diffuse polymorphous infiltrate to lymphoid, granulomatous, sclerosing, eosinophilic, or vasculitic inflammation.^14^

**Table 1.** Clinical Features.

	Case 1	Case 2	Case 3
Crohn’s diagnosis (age)	41	17	21
Orbital inflammatory disease presentation (age)	48	27	33
Symptoms	- Left lower eyelid swelling - Double vision	- Bilateral periorbital swelling - Pain - Double vision	- Left upper eyelid swelling
Signs	- Palpable orbital mass - Restricted extraocular motility - Globe elevation	- Left worse than right - S-shaped upper eyelid edema - Skin erythema - Restricted extraocular motility - Chemosis - Episcleritis	- S-shaped upper eyelid edema - Lobulated swelling of the lacrimal gland
Onset	Gradual (mo)	Acute (d)	Gradual (mo)
Previous immunosuppr.	6-mercaptopurine, adalimumab, methotrexate	Steroids, azathioprine, infliximab, adalimumab + methotrexate	Azathioprine, infliximab
Imaging	CT + MRI	CT	CT
Histopathology	Noncaseating granulomas (mass)	Vasculitis of medium-sized vessels and necrotizing histiocytic granulomas (lacrimal gland)	Noncaseating granulomas (lacrimal gland)
Treatment	Oral steroids	Oral steroids	Oral flurbiprofen

Abbreviations: CT, computed tomography; MRI, magnetic resonance imaging.

In our first and third cases, we observed unilateral orbital inflammatory changes. However, in case 2, bilateral orbital inflammation and a family history of vasculitis prompted an evaluation of systemic causes such as GPA, sarcoidosis, lymphoma, and IgG4-related disease. Although p-ANCAs were detected in case 2, patients with IBD are more likely to be p-ANCA positive than the general population, as are patients with idiopathic ocular inflammation with a family history of IBD.^15^ Perinuclear antineutrophil cytoplasmic antibodies are detected in 60% to 70% of ulcerative colitis cases, 10% to 15% of Crohn’s disease cases, and less than 5% of non-IBD colitis cases.^16,17^ Moreover, although histology demonstrated an acute necrotizing granulomatous vasculitis, this was not typical of a primary vasculitis and has been previously reported in OID.^14^ However, association between GPA and Crohn’s disease has previously been described,^18^ albeit in the absence of orbital involvement.

Pathophysiological mechanisms of EIMs in IBD are not clearly understood and warrant further study. Proposed pathogenetic autoimmune mechanisms include genetic susceptibility, antigenic display of autoantigen, aberrant self-recognition, and immunopathogenetic autoantibodies against organ-specific cellular antigens shared by colon and the extraintestinal organs.^19^ An immune response to a colonic antigen may explain why ocular manifestations occur more commonly with a colonic involvement.^20^ In cases 2 and 3, granulomatous dacryoadenitis may have arisen due to antigenic overlaps between gastrointestinal and lacrimal tissues, or alternatively, a gastrointestinal antigen may have localized hematogenously to the lacrimal glands’ location and incited a T-cell response, which produced an accompanying granulomatous reaction.^12^ In relation to genetic susceptibility, one study found that the prevalence of a family history of IBD is 3 to 15-fold higher in patients with orbital inflammation than the general population.^21^ Other studies have reported major histocompatibility complex associations with O-EIMs including human leucocyte antigen (HLA) haplotypes HLA-B27, B58, and HLA-DRB1*0103.^22^

Treatment of OID will depend on the extent of concomitant intestinal disease and additional EIMs. Although resolution may occur with observation alone or with antibiotics, corticosteroid treatment as seen in cases 1 and 2 generally results in much faster resolution of symptoms and is reserved for moderate-to-severe disease.^10^ Treatment of IBD-associated orbital inflammation can also be achieved with immunosuppression such as azathioprine and methotrexate and, when refractory, responds well to anti-tumor necrosis factor therapy.^2^ There are no reports on the efficacy of other licensed biological agents. It is therefore noteworthy that immunosuppression had been stopped in both cases 1 and 2 prior to developing orbital complications, which may have precipitated OID.

In conclusion, it is important to consider OID in the differential diagnosis of patients with Crohn’s disease, presenting with periorbital swelling and ocular motility dysfunction. Care should be taken to exclude orbital cellulitis, IgG4-related disease (with dacryoadenitis), lymphoproliferative and metastatic disease, sarcoidosis, and GPA. Patients presenting with ocular complications of their IBD should be managed through the combined care of gastroenterology and ophthalmology services where clinical evaluation should include careful history, physical examination, appropriate laboratory and radiographic orbital investigations, as well as need for diagnostic biopsy.
